# Draft Genome Sequence for the Type Strain of *Corynebacterium afermentans* LCDC 88-0199^T^, Isolated from a Human Blood Culture

**DOI:** 10.1128/genomeA.00661-16

**Published:** 2016-07-07

**Authors:** Anne-Marie Bernier, Kathryn Bernard

**Affiliations:** a Department of Biology, Université de Saint-Boniface, Winnipeg, Canada; b National Microbiology Laboratory, CSCHAH Site, Public Health Agency of Canada, Winnipeg, Canada; c Department of Medical Microbiology, University of Manitoba, Winnipeg, Canada

## Abstract

A draft genome for *Corynebacterium afermentans* LCDC 88-0199^T^ was investigated. The size of the genome was 2,345,615 bp with an observed G+C content of 64.85%. Annotation revealed 2 rRNA sequences, 54 tRNA genes, and 2,164 coding sequences. Genome coverage was 85× and consisted of 24 contigs with an *N*_50_ of 187,988 bp.

## GENOME ANNOUNCEMENT

*Corynebacterium afermentans* sp. nov., first described for Gram-positive bacilli designated by the CDC as absolute nonfermenter 1 (CDC group ANF-1) (1), was divided into *C. afermentans* subsp. *afermentans* and *C. afermentans* subsp. *lipophilum*, with both subspecies being essentially nonreactive to most phenotypic substrates but differing by *C. afermentans* subsp. *lipophilum* being lipophilic (2). Blood culture isolate LCDC 88-0199^T^, identified as CDC group ANF-1 by Health Canada’s Laboratory Centre for Disease Control (LCDC) in Ottawa, Canada (now the National Microbiology Laboratory in Winnipeg, Canada), was shared with French colleagues in the early 1990s and subsequently selected as the *C. afermentans* subsp. *afermentans* type strain (2). Here, we describe the draft genome of *Corynebacterium afermentans* LCDC 88-0199^T^ (= ATCC 51403T = CCUG 32103^T^ = CIP 103499T = DSM 44280T = JCM 10390T).

DNA from *C. afermentans* was purified using the TruSeq DNA HT sample preparation kit to prepare a paired-end whole-genome shotgun library, and sequencing was performed on the MiSeq Sequencer (Illumina 1.9, 2 × 300 cycles) according to manufacturer’s protocols, generating 1,265,540 reads and 379,796,318 detected bases, for an average estimated read coverage of 161×. Overlapping paired-end reads were merged using FLASH (3) to produce longer single reads that were subsequently assembled with paired-end reads using SPAdes genome assembler (St. Petersburg genome assembler, version 3.5.0) (4) using *k*-mer values of 21 to 127. This generated 71 contigs, which were subsequently filtered to remove repeated sequences and sequences <1 kb, leaving 24 contigs with an *N*_50_ of 1,870,988 bp. The average contig size was 97,733 bp with an average coverage of 85×.

Gene prediction and annotation was performed with the NCBI Prokaryotic Genome Annotation Pipeline (http://www.ncbi.nlm.nih.gov/genome/annotation_prok), which revealed 5 rRNA genes, 52 tRNA genes, 51 pseudogenes, 2,197 genes, and 2,137 coding sequences. One clustered regularly interspaced short palindromic repeat sequence was found. The genome size of *C. afermentans* was 2,345,615 bp with a G+C content of 64.85%, which is comparable to the 66% G+C content obtained using high-performance liquid chromatography methods (2). Blast analysis using a locally constructed database from publically available sequences from *C. glutamicum* ATCC 13032^T^ and *C. diphtheriae* NCTC 13129 revealed that the *C. afermentans* genome encoded for mycolate synthesis proteins, with identity ranging between 51% and 76% among proteins examined (data not shown) (5). Such data are consistent with the expected presence of mycolates. A fatty acid synthase (FAS) gene was also detected by *in silico* analysis, which is consistent with the nonlipophilic phenotype of *C. afermentans*. No phages were found in the genome using PHAST (http://phast.wishartlab.com) (6). LCDC 89-0199^T^, along with the other 8 *C. afermentans* isolates referred to here, has been described as being multidrug resistant (7). A homolog to the *C. urealyticum* ermX gene was found (93% protein identity), confirming the published resistance to clindamycin and erythromycin (7).

### Nucleotide sequence accession number.

The draft whole-genome project for *C. afermentans* subsp. *afermentans* LCDC 89-0199^T^ has been deposited at DDBJ/EMBL/GenBank under the accession number LXGG00000000.
